# Cellular-scale probes enable stable chronic subsecond monitoring of dopamine neurochemicals in a rodent model

**DOI:** 10.1038/s42003-018-0147-y

**Published:** 2018-09-12

**Authors:** Helen N. Schwerdt, Elizabeth Zhang, Min Jung Kim, Tomoko Yoshida, Lauren Stanwicks, Satoko Amemori, Huseyin E. Dagdeviren, Robert Langer, Michael J. Cima, Ann M. Graybiel

**Affiliations:** 1 McGovern Institute for Brain Research and Department of Brain and Cognitive Sciences, Massachusetts Institute of Technology, Cambridge, MA 02139 USA; 2 Koch Institute for Integrative Cancer Research, Massachusetts Institute of Technology, Cambridge, MA 02139 USA; 3 Department of Chemical Engineering, Massachusetts Institute of Technology, Cambridge, MA 02139 USA; 4 Department of Materials Science and Engineering, Massachusetts Institute of Technology, Cambridge, MA 02139 USA

## Abstract

Chemical signaling underlies both temporally phasic and extended activity in the brain. Phasic activity can be monitored by implanted sensors, but chronic recording of such chemical signals has been difficult because the capacity to measure them degrades over time. This degradation has been attributed to tissue damage progressively produced by the sensors and failure of the sensors themselves. We report methods that surmount these problems through the development of sensors having diameters as small as individual neuronal cell bodies (<10 µm). These micro-invasive probes (µIPs) markedly reduced expression of detectable markers of inflammation and tissue damage in a rodent test model. The chronically implanted µIPs provided stable operation in monitoring sub-second fluctuations in stimulation-evoked dopamine in anesthetized rats for over a year. These findings demonstrate that monitoring of chemical activity patterns in the brain over at least year-long periods, long a goal of both basic and clinical neuroscience, is achievable.

## Abstract (web summary)

Helen Schwerdt et al. report micro-invasive probes capable of monitoring chemical signaling in the rat brain for over a year. The probes have diameters as small as single neuronal cell bodies and can monitor sub-second fluctuations in chemical signaling without significant induction of inflammation or tissue damage.

## Introduction

Prolonged changes in chemical signaling and neurotransmission in the brain are thought to underlie cognition, learning, and fluctuations in mental state. Methods for long-term, stable monitoring of the molecular status of individual brain cells, circuits and networks are particularly critical. With these, advances could be made not only in understanding the normal functions of molecular species over extended time-periods, but also in studying the etiology of debilitating neural disorders^1–3^. A major test case for chronic application of neurochemical sensors has been in the use of microscale carbon fiber electrodes (CFEs) to detect redox current of dopamine by fast-scan cyclic voltammetry (FSCV)^4^. Implantable interfaces have enabled measurement of dopamine at highly localized (microns) and rapid (milliseconds) dynamic scales at which these molecules operate in the brains of rodents and primates^4–17^. The capacity of currently existing neurochemical sensors to provide reliable signals over extended time-frames approaching a year has, however, been extremely difficult to achieve because the signals degrade over time-frames tested^6,12,18^. Neurochemical sensors have displayed consistent decline of the signal amplitude over time^6^ and temporal distortion (e.g., lengthened changes in computed dopamine release)^12^. This apparent degradation brings into question the accuracy of extended measurements and thus lessens the possibility of tracking dopamine over slowly developing behaviors and disease conditions, including Parkinson’s disease.

Here, we attempted to resolve these problems in order to provide at least year-long accurate measurements of dopamine release in rats. We first built on our initial attempt^15^ to create micro-invasive probes (µIPs) to address these barriers to chronic implantation. A 7 µm diameter carbon fiber is normally used as it has been shown to provide the optimal electrochemical sensing interface for sensitive detection of dopamine and other electroactive compounds^4–17^. Furthermore, the cellular-scale carbon fiber is small enough on its own to produce negligible perturbation and tissue response in the brain^15,19,20^. Chronically applied CFEs, typically constructed with a 90 µm diameter silica shaft^12^, occupy implant footprints large enough to disrupt normal neuronal circuitry and astrocytic configurations that can extend up to 150 µm and farther from their implanted path and structure^20–24^. These inflammatory processes could thus alter extracellular chemical gradients and lessen the physiologic accuracy of sampled molecules at the carbon fiber sensing interface^25,26^. The induced trauma further limits practical, safe use in humans. The µIPs that we developed have cross-sectional diameters on the order of individual neuronal cell bodies (<10 µm diameter)^15^, which are the smallest of any implantable neurochemical sensor available. These sensors gave accurate signals and minimum tissue damage, but were not tested for long-term use. Here, we report modifications of the sensors and their implantation that allow stable, reliable dopamine monitoring for periods of over a year, promising for clinical application.

## Results

### Probe optimization for extended chronic performance

**Fig. 1:**
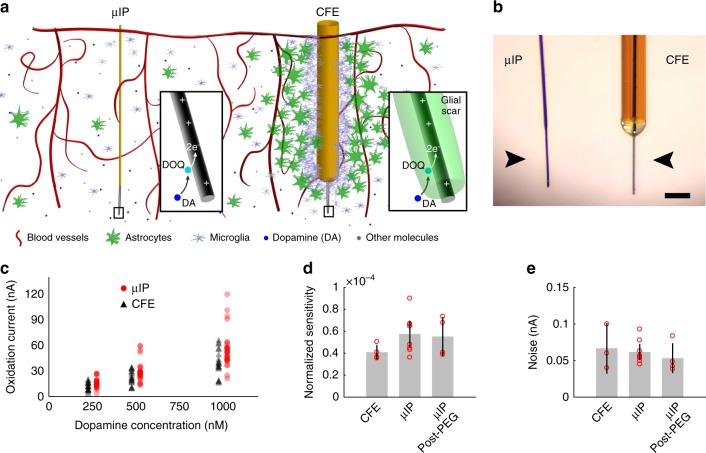
Probe operation and performance in neurochemical recording. **a** Schematic illustration of cellular-scale µIP and CFE implanted in striatum. Insets: close-up of µIP and carbon fiber sensor tips demonstrating potential restricted diffusion of dopamine (DA) molecules through inflammatory scar tissue as they reach a positively charged carbon fiber to oxidize, forming dopamine-o-quinone (DOQ), and transferring 2 electrons (e^−^) into the carbon fiber for current measurement. **b** Photograph of µIP and CFE (arrowheads point to center of sensing tips). Scale bar: 100 µm. **c** In vitro measurements of dopamine oxidation current recorded as a function of dopamine concentration for µIP and CFE. **d** Normalized sensitivity (nA nM^–1^ nA^–1^) of µIP (before and after PEG coating) and CFE, showing no differences for probe types or effects of PEG deposition. **e** Noise level of µIP and CFE, showing no differences for probe types or PEG application. Error bars represent 95% confidence intervals (i.e., ±SEM ×1.96)

We designed controlled coating and etching methods that allowed very long-term recording (Fig. 1a, b). A thin layer (0.7–1.3 µm) of parylene-C, an FDA-approved biocompatible (USP Class VI) dielectric^27^, was applied onto bare carbon fiber (7 µm diameter and 5–7 mm long) to provide a highly conformal and impermeable insulation (Supplementary Fig. 1). A discrete 50–200 µm length of carbon fiber was exposed at the tip to produce the active chemical sensing interface^10,16,17^ by controlled etching of the parylene. The other end of the probe was conductively bonded to metal interconnects on a printed circuit board to transfer current acquired from the carbon fiber sensor to FSCV instrumentation for chemical recording.

Exposing the carbon fiber sensing surface was the most critical fabrication step in determining final functional stability. Reproducible thermal etching procedures were implemented to generate chronically stable interfaces. We compared these thermal patterning procedures to the traditional lift-off techniques. Flame-etching produced a smooth boundary between parylene and the exposed carbon fiber^15,28^, but could be difficult to control. A heated micro-coil could be placed at individual probe tips to yield reproducible stripping. Both types of heat treatment, moreover, could enhance the carbon fiber sensitivity to dopamine, as it generates more functional oxygen-containing groups at the carbon fiber surface, thus accelerating electron transfer and the adsorption of cations^16^. Lift-off techniques were also explored for patterning. Lift-off allowed high throughput patterning of the carbon fiber tips across an array of probes, and probes patterned in this manner were successful in recording dopamine in acute, single-use, in vivo recording experiments^15^. Lift-off, however, requires peeling the conformal parylene away from the carbon fiber substrate, producing mechanical stress that could compromise the adhesion between these two materials. These effects did not impair acute measurements, but some lift-off patterned probes exhibited increased noise over extended voltammetric operation (Supplementary Fig. 2)^29,30^. Thermal patterning techniques therefore were selected to maximize stability of the probes for long-term performance.

The resulting µIPs have a maximum cross-sectional area of ~60 µm^2^, which is over 100 times smaller than that of CFEs (~6362 µm^2^) built from fused-silica capillaries (90 µm diameter)^12^. The smaller size resulted in a flexural rigidity that is over three orders of magnitude lower than that of CFEs (µIP: *K* < 8 × 10^−11^ N m^2^; CFE: *K* > 2.3 × 10^−7^ N m^2^). The small areal extent of the implanted µIPs minimized tissue response^19,31^, while the resulting increased compliance attenuates micromotion-induced inflammation of the indwelling implant^32,33^ (Fig. 1a, b). The small size and flexibility enabled by the thin film parylene, in combination with the thermal patterning technique, had the critical function of establishing the long-term performance of the µIPs.

The extremely small and flexible features of the µIPs required temporary encasement in a rigid polyethylene glycol (PEG) shuttle (0.5–1 mm thick) (Supplementary Fig. 1). This mechanically transient shuttle provided the necessary stiffness to assist with subsequent brain insertion in rats. PEG was selected for the shuttle material because of its biodegradability and immunologically inert characteristics^34^, as well as its ability to resist protein adsorption and cellular adhesion^35,36^. The PEG shuttle was incrementally dissolved just above the brain surface, so as to suspend small lengths of the probes as they were progressively lowered without deflection into the tissue. This procedure preserved the cellular-scale dimensions (<10 µm diameter) and compliance of all implanted parts of the devices.

### In vitro functional characterization

The probes were characterized in vitro to ensure that their chemical detection capabilities conformed to that of CFEs, that performance was unaffected by PEG coating, and that stable operation could be demonstrated during continuous use in artificial cerebrospinal fluid (aCSF). The µIPs and CFEs were tested, in parallel, in a flow cell containing aCSF to determine how responsive they were to physiologic concentrations of dopamine (i.e., sensitivity). Overall background noise levels (Methods) were also measured to determine the minimum distinguishable concentrations (i.e., limit of detection, LOD) of the sensors. We used FSCV in all reported measurements to record electrochemical current generated at the carbon fiber in response to the reduction and oxidation (redox) of dopamine^10,18,37^. The cyclic voltammograms recorded by both types of probe conferred selective dopamine measurement based on the generation of distinct oxidation and reduction current peaks at, respectively, ~0.6 and −0.2 V, which correspond to the dopamine redox potentials^10,18,37^. Both µIPs and CFEs produced oxidation current correlated linearly with dopamine concentrations within a tested physiological range of 0.25–1 µM (Pearson’s *R* > 0.99) (Fig. 1c and Supplementary Figs. 3, 4a).

Dopamine oxidation is directly proportional to the background capacitive current, which is representative of the active area of the carbon fiber chemical sensing interface^15^. The measured oxidation current was normalized to the apparent capacitance of the sensor to account for differences in the final exposed lengths of carbon fiber across probes. The normalized sensitivity was virtually unchanged (*P* = 0.41, two-tailed *t*-test) for both µIPs (5.75 ± 1.22 × 10^–5^ nA nM^–1^ nA^–1^) and CFEs (4.09 ± 0.67 × 10^–5^ nA nM^–1^ nA^–1^) (Fig. 1d and Supplementary Fig. 4b, c). The current noise of the µIPs and CFEs was also nearly the same (*P* = 0.64, two-tailed *t*-test; µIP: 0.10 ± 0.10 nA, and CFE: 0.07 ± 0.03 nA) (Fig. 1e).

The effects of applying biodegradable PEG to shuttle the µIPs during implantation in rats (Supplementary Fig. 5) were evaluated to determine whether application could compromise dopamine measurement performance. The sensitivity of the probes was not significantly different (*P* = 0.92, two-tailed *t*-test) before treatment (5.75 ± 1.22 × 10^–5^ nA nM^–1^ nA^–1^) and after PEG treatment (5.52 ± 1.8 × 10^–5^ nA nM^–1^ nA^–1^) (Fig. 1d). The noise was also unchanged (*P* = 0.40, two-tailed *t*-test) before (0.06 ± 0.01 nA) and after PEG application (0.05 ± 0.01 nA) (Fig. 1e). These measurements provided evidence that the µIPs retain comparably low noise, nanomolar sensitivity, and LOD as the larger CFE sensors, and that this performance is unaffected by the deposition of dissolvable PEG materials.

### Tissue response characterization

Obstructed transport of targeted molecules to implanted CFE chemical sensing interfaces has been hypothesized to underlie much of the decline in capacity of chronic chemical recordings^6,12^. Our goal in creating smaller implants was to reduce the induced inflammatory response and tissue damage surrounding the probes, which can restrict neurochemical diffusion^6,12^ and can compromise the long-term safety of the implant. We compared tissue response surrounding the implanted shafts of both µIPs (<10 µm diameter) and CFEs (90 µm diameter) after 74–361 days of chronic implantation in the brains of rats. This assessment was performed around the shafts to evaluate the trauma representative of the majority of the implanted device. The histochemical analyses were performed on successive horizontal brain sections to identify the vertical tracks of implanted devices through the tissue of the striatum. Selected sections were ~0.5–1 mm away from the probe tip and >2 mm from the dural surface to focus on regions around shafts, away from lesioned tips and away from any surgically induced trauma at the cortical surface. Selective markers of activated microglia (Iba1), astrocytes (glial fibrillary acidic protein, GFAP), and blood brain barrier permeation (immunoglobulin G, IgG) were used to assess the tissue inflammatory response (Supplementary Fig. 6), with the contralateral striatum as control tissue.

The µIPs induced remarkably negligible tissue response, in contrast to the response to CFEs (Supplementary Figs. 6b–d and 7). The peak fluorescence intensity observed in µIP-implanted regions was 1.52 for GFAP, 1.17 for IgG, and 1.65 for Iba1, relative to background levels (taken 600 µm from probe origin). The astrocytic and microglial responses were not significantly different in comparison to unpenetrated regions of tissue (*P* = 0.2653 for GFAP and *P* = 0.9547 for Iba1, two-tailed *t*-test). The CFEs produced extensive responses of 6.73 for GFAP and 10.5 for IgG (*P* < 0.01, two-tailed *t*-test). The minimal neuroimmunological response induced by µIPs could provide less restricted diffusion of targeted dopamine molecules to the sensor, thus reducing temporal distortion, a well-known limitation of CFE-based dopamine measurements^12,38^.

Tissue disruption was also assessed by evaluating the displacement of cell bodies stained by 4’,6-diamidino-2-phenylindole (DAPI) around the center of implanted sites. The radius of this protrusion was measured by computing the distance from the center of the implant track to the position at which DAPI intensity reached background levels. µIPs displaced cells to a radius of 5 µm, whereas CFEs induced 34 µm of displacement. These values correspond closely to the radius of the implanted shaft, as expected. The negligible tissue distortion induced by the µIP could help to retain normal extracellular chemical signaling processes, thus promoting more accurate measurements of neuronal circuit activity.

The reduced expression of inflammatory markers, coupled with the smaller disruption of the local cellular infrastructure, illustrates the capacity of the µIPs to induce minimal damage to healthy tissue of the nervous system. We next tested whether these properties could enhance their performance in up to year-long molecular sampling.

### Longitudinal dopamine measurements at chronic timescales

**Fig. 2:**
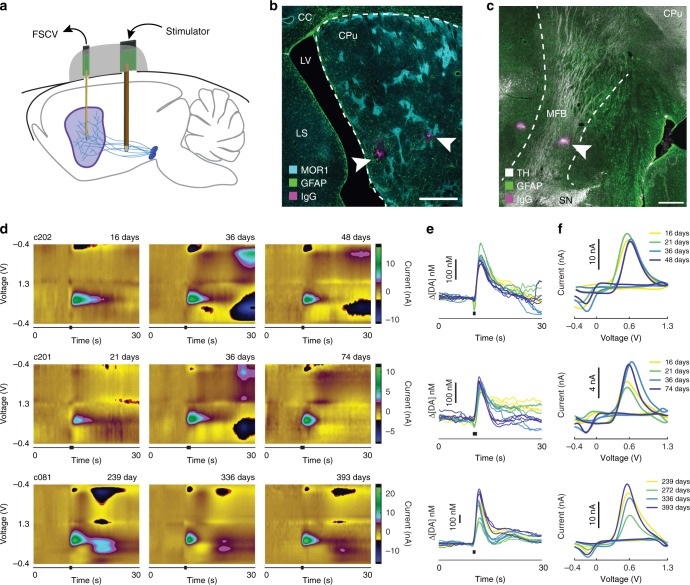
Dopamine release measurements acquired by chronically implanted probes. **a** Experimental setup for long-term testing in rats. **b**, **c** Photomicrographs of horizontal sections showing implanted sites for recording in striatum including site in vicinity of striosomes expressing mu-opioid receptor 1 (MOR1) (**b**) and stimulation site within MFB, shown by immunostaining for tyrosine hydroxylase (TH) (**c**). Arrowheads point to lesion sites marking recording or stimulation locations. Dashed lines indicate approximate boundaries of targeted implantation regions. CPu caudoputamen, LS lateral septum, LV lateral ventricle, CC corpus callosum, SN substantia nigra, and MFB medial forebrain bundle. Scale bars represent 500 µm. **d**–**f** Representative dopamine measurements from three µIPs (c202, c201, and c081) in two rats across several time points in the form of background-subtracted color plots from three different days (**d**), the extracted dopamine concentrations (Δ[DA]) vs. time traces (**e**) and the cyclic voltammograms (**f**) from four different days. Stimulation is uniform for each time point for a given probe, and its time is indicated by black bars below color plots and Δ[DA] vs. time traces

We evaluated the capacity of the µIPs to provide reproducible measurements of dopamine at multiple chronic time points from 14 to 398 days postimplant in anesthetized rats. In each rat (*n* = 7), 3–5 µIPs were implanted into the striatum in order to increase the yield of implanted sites situated in active dopamine-releasing volumes (~50% probability)^15^ (Fig. 2a, b and Supplementary Fig. 5). The dissolvable PEG shuttle allowed linear insertion of its encased probes into the deep-lying striatal tissue (~4–5.5 mm depth). Saline was applied to the brain surface to dissolve the lower exposed parts of the shuttle prior to the insertion of its encased probes. This step proved to be critical in preventing the insertion of the larger, rigid shuttle, which otherwise produced permanent tissue damage (Supplementary Fig. 7c). Successful targeting of probes to the striatum was confirmed by making final electrolytic lesions at selected probe tips followed by histological examination (Fig. 2b). Stimulation electrodes were implanted into the ipsilateral medial forebrain bundle (MFB) to evoke striatal dopamine release controllably (Fig. 2c). Measurements were made from the implanted probes for days to weeks after their surgical implantation.

Recordings with the µIPs demonstrated successful measurement of dopamine release at extended time-points after implantation (Fig. 2d–f and Supplementary Fig. 8a, b). Initial recording performed across several days indicated selective dopamine redox current, and these redox curves (i.e., cyclic voltammograms) were consistent among recordings (Fig. 2d–f). The temporal dynamics of the measured dopamine release was also retained over time (Fig. 2d–f). These signals were also comparable to those recorded from acutely implanted probes (Supplementary Fig. 8c).

**Fig. 3:**
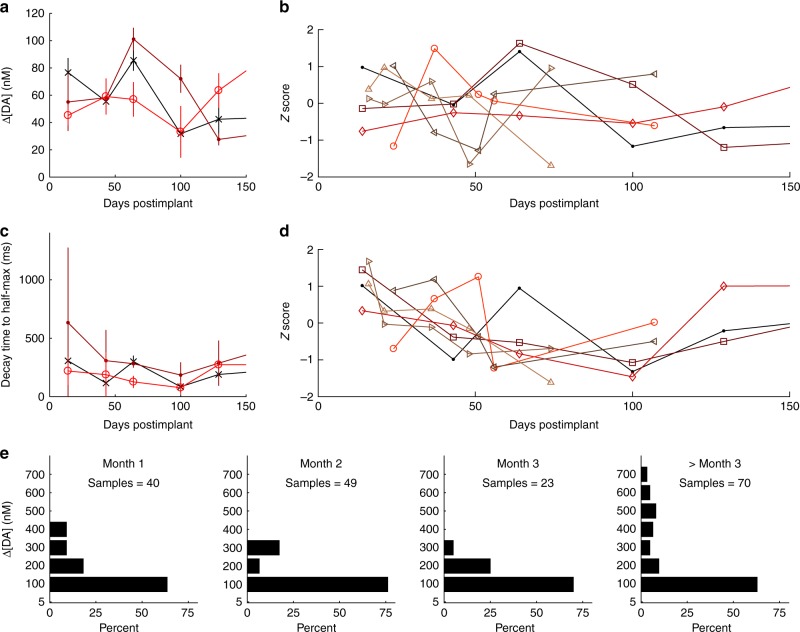
Chronically implanted µIPs provide reliable longitudinal measurement over a wide range of dopamine concentrations and over the course of months postimplant. **a** Measured dopamine (DA) over time from three different probes, c121 (**×**), c122 (•), and c123 (**○**), in a single rat for a fixed stimulation parameter. **b** Recorded dopamine concentrations across time normalized to show fluctuations in measured dopamine release for each of 7 probes, c121 (•), c122 (**⎕**), c123 (◊), c171 (**○**), c172 (⊲), c201 (⊳), and c202 (▵), implanted in three rats. Stimulation parameters were fixed for each rat across measurement sessions. **c** Measured decay time from maximum to half-maximum evoked dopamine for same probes and parameters as in **a**. **d** Normalized decay times for same parameters and probes as in **b**. **e** Binned recorded dopamine concentrations detected by all functional probes (eight probes in four rats) and for the fixed stimulation parameters as in **b** and **d**, plotted by month. Error bars represent 95% confidence intervals

The longitudinal sustainability and reproducibility of chemical measurements made from the implanted µIPs were examined over days to months (5–10 measurement sessions, each from eight probes across four rats). A constant set of stimulation parameters was applied to each rat in an attempt to fix the levels of striatal dopamine overflow sampled by the implanted probe (Supplementary Data 1). Consistent amplitudes of dopamine were measured from all of the implanted µIPs with repeated stimulation in a given session (average standard deviation of 18.1363 nM for *n* = 26 recording sessions). These amplitudes, however, varied from day to day (Fig. 3a and Supplementary Fig. 9a–d).

The µIPs individually displayed both increase and decrease in recorded dopamine, but these changes were not correlated with elapsed time since implantation or with implanted subject (Table 1). The trend of measured dopamine across all implanted probes was computed to evaluate whether the changes in evoked concentrations followed any consistent increasing or decreasing trend (Table 1). These trends were uncorrelated with elapsed time (Pearson’s *R* = 0.1304–0.8992 as measured from eight probes implanted across four rats), indicating the lack of a significant relationship with the indwelling periods of the implants. Equal levels of increasing and decreasing trends were observed from the chronically implanted probes, and both types of trends were observed from multiple probes implanted within an individual subject, affirming that these trends were not related to the specific subject.

**Table 1:** Trends of longitudinal changes in dopamine measured with chronically implanted µIPs

Rat ID	Average DA for each rat (nM)	Standard deviation (nM)	Probe ID	Average DA for each probe (nM)	Standard deviation (nM)	Over time trend (nM/day)	Over time *R*	*P*-value
A	446	n/a	c081	446	138	1.5	0.7093	0.1797
B	60	2.5	c121	57	20	−0.17	0.61	0.2022
B			c122	59	26	−0.17	0.48	0.3369
B			c123	63	13	0.34	0.7875	0.0630
C	39	1.4	c171	40	18	−0.10	0.1826	0.7688
C			c172	38	9.2	0.07	0.2331	0.7059
D	179	73.5	c201	127	50.2	0.28	0.1304	0.8344
D			c202	231	125	−4.82	0.8992	0.0378

**Fig. 4:**
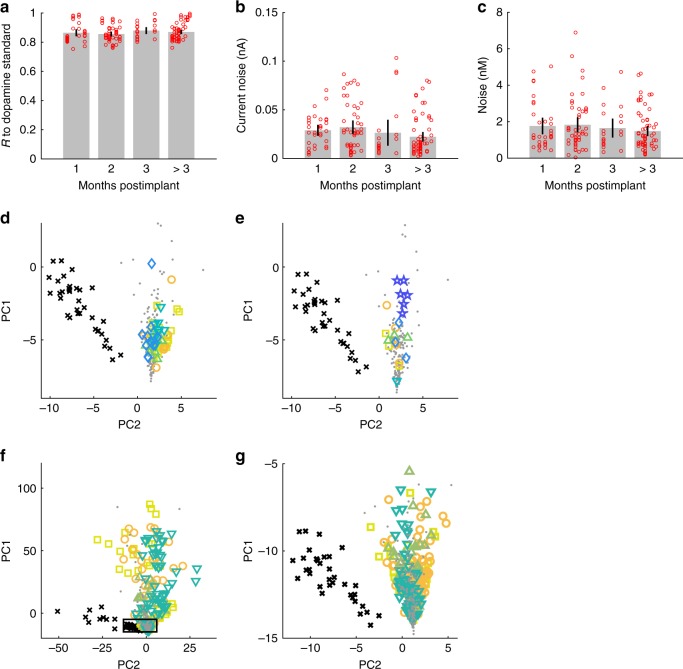
Long-term performance of measurements by µIPs and fidelity of detected signals. **a** Pearson’s correlation coefficients (*R*) of cyclic voltammograms recorded in vivo to in vitro dopamine standards, computed from 188 recordings from 8 probes in four rats at all chronic time points and for the same stimulation parameters as in Fig. 3e, plotted over periods of 1, 2, 3, and >3 months postimplant. *R* > 0.75 for all chronically recorded signals, and these correlations are maintained over time. **b** Current noise of measured signals. **c** Dopamine concentration noise of measured signals (as extracted from data presented in **b** by PCA) demonstrating an average LOD of 5.7 nM (mean noise × 3). Error bars represent 95% confidence intervals. **d**–**g** Principal component scores (PC1 and PC2) of recorded in vivo signals (colored shape signals; same data source as presented in **a**–**c**) as measured at 24 (⎕), 37 (**○**), 51 (▵), 56 (∇), and 107 days (◊) postimplant for probe c172 (**d**), and at 14 (⎕), 43 (**○**), 64 (Δ), 100 (∇), 129 (◊), and 212 days (☆) postimplant for probe c123 (**e**), and at 1 (⎕), 2 (**○**), 3 (Δ), and greater than 3 months (∇) for all implanted probes (eight probes in four rats) (**f**). Measurements are overlaid with scores from dopamine (•) and pH (×) standards as taken from 96 in vitro measurements from three probes. In vivo principal component scores demonstrate tight correspondence between measured cyclic voltammograms and the dopamine standards with a clear separation from the pH cyclic voltammograms, indicating clear distinction to pH interferants. The close-up (**g**) of the outlined area in **f** shows separation between group of pH scores and all measured cyclic voltammograms that closely overlay with the dopamine standards

Measured dopamine (DA) averaged across all recorded evoked signals from all chronic time points for each rat or for individual probes. Stimulation parameters are same as those implemented in Figs. 3e and 4a–c. Trend computed by taking the slope of a line fitted to recorded DA as a function of days. The *R*-value is Pearson’s correlation coefficient between measured DA and days, and its *P-*value tests the significance of *R* against a value of 0

The levels of recorded dopamine were specific to the individual animal, as the average concentration of dopamine was significantly different among the rats (*P* < 0.0001, one-way ANOVA for all four rats), but not across probes within a given rat (*P* = 0.1232–0.9333, one-way ANOVA computed from 2–3 probes in each of three rats). This variability could be related to slight differences in position of the stimulating electrode relative to the topographic MFB connections to the striatal recording sites. The standard score (*Z*-score) of changes in evoked dopamine was calculated to assess variability over time (Fig. 3b) to normalize the scales of recorded concentrations that varied widely across subjects. The intensity of the MFB stimulation was also adjusted to tune striatal dopamine overflow by varying its duration and/or amplitude^4–6,8,9,12,39^. Increased stimulation intensities consistently amplified dopamine release (Supplementary Fig. 8b).

Temporal dynamics of evoked dopamine signals was also computed to characterize the capacity of implanted µIPs to retain subsecond resolution (Fig. 3c, d and Supplementary Fig. 9e–h). The average decay time to half maximum of the peak evoked dopamine concentration, across all probes from chronic measurement sessions, was 119 ms. Day-to-day changes in decay times were not significant and were not correlated with recorded time-points for most probes (6 out of 8). Significant decreases were found in two of the implanted probes in a single rat, and three of the probes exhibited downward trends correlated with duration of implantation (Pearson’s *R* = 0.89, 0.75, and 0.96 for c172, c201, and c202, respectively). These variations and trends were most significant during the first 2 months of indwelling and could be related to an early phase of inflammation induced by acute trauma following surgical implantation^20,31^, or to differences in activation of downstream MFB structures.

We aggregated all of the evoked signals recorded each month from the implanted probes (for a fixed stimulation parameter per probe) to allow a comprehensive assessment of the distribution of concentrations sampled by the µIPs (Fig. 3e). The probes reproducibly captured a wide range of dopamine concentrations from month to month, collectively measuring ranges of up to 600 nM in the first month, and up to 700 nM at all periods >3 months postimplant.

### Sustained fidelity of neurochemical measurements

To estimate the inherent capability of the implanted µIPs to exhibit nanomolar sensitivity and dopamine chemical-specific measurements, we computed metrics of selectivity and LOD across successive recorded sessions. Chemical specificity was conferred by the appearance of peak current changes at the dopamine redox potentials (i.e., –0.2 and 0.6 V) of measured cyclic voltammograms, as well as by their correlation to dopamine standards obtained in vitro. All chronically measured cyclic voltammograms expressed distinctive dopamine redox potentials and were highly correlated with those of dopamine standards (Pearson’s *R* = 0.87). These strong correlations were reproducibly observed across successive measurements made over months (Fig. 4a).

The average background current noise from all chronic measurements was 0.04 nA with an equivalent dopamine concentration noise of 1.9 nM (i.e., after extraction of projected dopamine concentration by principal component analysis [PCA]). The noise value represents the practical LOD of the probes: a signal-to-noise ratio of ≥3 is a frequently applied criterion implemented for voltammetric dopamine measurements^37^. The noise level is also representative of the implant’s structural integrity. A perforated or delaminated insulation and/or increased resistance in the measurement circuit produced by mechanical breakage along the carbon fiber or its junction to the printed circuit board could increase random current fluctuations. The low values for the µIPs were not significantly variable from month to month or for periods of greater than three months (*P* > 0.05, two-tailed *t*-test) (Fig. 4b), and were not significantly different from in vitro measurements (*P* > 0.05, two-tailed *t*-test) (Fig. 1e).

Changes in pH are considered to be a major source of interference for electrochemical recording. pH fluctuations induce currents over a broad range of voltages, obscuring the current changes at the targeted dopamine redox potentials^7,11,40^. PCA was used to characterize how well the recorded signals could dissociate dopamine from pH. Cyclic voltammograms recorded in vivo were combined with cyclic voltammograms of in vitro dopamine and pH standards, and were represented in the principal component space (i.e., scores) (Fig. 4c–e). The in vitro principal component scores for dopamine (gray dots in Fig. 4d, e) and pH (black crosses in Fig. 4d, e) standards formed two well-defined clusters, which confirmed selective discrimination of these two chemical species as recorded by the µIPs in a controlled flow-cell environment. The in vivo cyclic voltammograms, acquired from single chronically implanted probes during MFB stimulation, overlapped tightly with the cluster of scores for dopamine standards (Fig. 4d, e and Supplementary Fig. 10). PCA was applied to all chronic in vivo measurements from successfully implanted µIPs together with the in vitro dopamine and pH measurements. Principal component scores for all chronic in vivo µIP measurements formed a tight feature space that overlaid the dopamine standards and were clearly separable from the pH cluster (Fig. 4f).

## Discussion

This study introduces, to our knowledge, the first sensor capable of monitoring chemical activity in the brain over year-long time periods. We tested our probes in anesthetized rats using controlled electrical stimulation to assess performance in dopamine chemical monitoring over time. There has been very little prior information about the fidelity of such chronically implanted chemical sensors as a function of time^6^. Here, we approached this issue quantitatively. We implemented longitudinal metrics of probe integrity in terms of noise, selectivity, and correlation of the recorded signals to in vitro standards. Our findings not only demonstrate the chronic recording capability of the µIPs, but further corroborate µIP function in recording subsecond dopamine dynamics with preserved sensitivity, accuracy, and selectivity without significant infraction of the tissue environment. The sustained performance of the cellular-scale neurochemical µIPs validates their use in recording rapid chemical fluctuations with the needed reliability over many months.

Sensors so far have not been shown to record chemicals without degradation of signals over time. The protocols reported here were developed to quantify comprehensively signal stability and longitudinal performance, parameters critical to ensure measurement accuracy of the implanted chemical sensors. Decreasing both the diameter and the stiffness of the implanted devices could be critical to reducing disruption of the submicron cellular architecture that underlies neurotransmission. Very few differences were observed in the relative intensity of inflammatory markers between intact brain tissue and µIP implanted sites examined in the same brain in ipsilateral and contralateral hemispheres. CFE chemical sensor implants tended to cavitate the tissue and to create widespread damage in the form of scarring, inflammation, blood brain barrier disruption, and infiltration of foreign molecules and toxins. Tissue responses were measured around the implanted shafts 0.5–1 mm dorsal to their sensing tips to evaluate contributions of the bulk of the implant, and these shafts differentiated the geometry of the µIP from the CFE implant. Shaft-induced tissue responses were observed to spread over hundreds of micrometers—distances sufficient to encapsulate some portion of the sensing tip that would restrict diffusion of targeted molecules to the sensing interface and undermine long-term function. In addition to implant size, other factors, including the mechanical and/or material properties of the implanted shaft, could have distinctive influences on tissue response and the progressive performance of neurochemical measurements^20,31^. The cellular-scale probes reported here markedly minimized tissue disruption and inflammation, which could have helped enable longitudinally stable recording of neurochemicals over chronic timescales.

The chronically implanted µIPs detected a wide range of dopamine concentrations as evoked by controlled electrical stimulation. We expected a consistent decrease in the amplitudes of detected dopamine, as seen before with CFEs^6^ in what remains, to our knowledge, the only existing report of recording stimulation-evoked dopamine over time. Instead, we found that the µIPs collectively displayed no coherent trend in detected dopamine levels, and the cumulative distributions of recorded concentrations were similar from month to month. The lack of a decreasing trend in recorded signals and the similar range of detected concentrations across probes demonstrate the sustained sensitivity and accuracy of the chronically implanted µIPs.

The concentrations recorded by the chronic probes did, however, vary from day to day. These variations could be due to changes in the probe’s sensitivity, movement of striatal probes or MFB stimulation electrodes, depth of anesthesia^14^, and/or changes in stimulation efficacy^4,39^. Microscale differences in the location of the stimulation electrode in the MFB can significantly affect the amount of dopamine released at its projections within the striatum^13,15^. This variability could result from greater intra-tissue movement, and induced tissue response^39^, of the stimulation electrode, which is over 200 times larger (cross-sectional area of 12,272 µm^2^) and therefore 196 times stiffer (axial stiffness of ~374 kN/m) than the striatal µIP. Further, we have not demonstrated here the operation of the µIPs in awake, behaving animals, in which it is likely that day-to-day variations in maximum concentration detected by these probes could be higher based on the state of the animal. Methods to confer uniform detection environments and sensitivities of the implanted probes will thus be needed to make accurate comparisons of amplitude fluctuations between sessions. Nevertheless, an encouraging property of the chronic measurements made with the µIPs is the sustained temporal characteristics of the recorded dopamine signals. This feature could have important consequences in accurately characterizing the wide range of temporal signaling dynamics of dopamine and other neurotransmitters and neuromodulators.

Cellular-sized implants have rapidly emerged over the last few years with the goal of alleviating tissue response and enabling chronic recording of electrical neural activity^19,41–43^. The chronically operational devices that we report here allow tracking of chemical activity for periods up to a year, durations suitable for analyzing dopamine release related to learning and to changes in motivational states in animals. Future experiments in non-human primates over even longer periods of recording will be needed to assess the feasibility of the use of these sensors in humans. If successful, such an ability to perform chronic neurochemical measurements could be critical in improving and enabling online diagnostics for adaptive remediation of debilitating conditions, including Parkinson’s disease and other movement and mood disorders.

## Methods

### Probe fabrication

Probes (8–10 µm maximal diameter) were fabricated largely as previously reported^15^ (Supplementary Fig. 1). Individual carbon fibers having diameters of 7 µm (Goodfellow, C 005722) and cut to lengths of ~10 mm were bonded to a printed circuit board with conductive traces that coupled to a connector socket (Mill-Max, 853-93-100) using silver epoxy (Epo-Tek, H20S). A glass micromold with 80 µm wide trenches (Precision Micro-Optics, PGVG-1082205) was used to keep individual carbon fibers aligned on the board and separated from each other. The silver epoxy was cured by heating at 80–120 °C for 2–8 h. Devices were cleaned by rinsing with isopropanol and then immersed in an adhesion promoter solution consisting of A174 (Sigma-Aldrich, 440159), isopropanol, and distilled water at a volumetric ratio of 1:100:100 for 10–15 min. The devices were dried in air, rinsed again in isopropanol, and then thoroughly dried. Parylene was coated uniformly over all devices at a thickness of 0.7–1.3 µm (Specialty Coating Systems, PDS 2010 Labcoater). An open flame from a butane torch (Weller, P2KCKIT) was used to expose carbon fiber sensing sites at the probe tips for the majority of implanted probes. Excessive heat could warp the parylene, and therefore the body of the probes was thermally insulated in water. Bare carbon fibers were further trimmed to lengths of 50–200 µm with fine scissors if they were too long after thermal etching. Equivalent performance of the subcellular µIPs was confirmed with CFE sensors in vitro (Fig. 1c–e and Supplementary Figs. 3 and 4)^15^. The electrical current produced in response to various physiological concentrations of dopamine was virtually identical for both types of sensor. The noise level of both sensor types was also comparable. These maintained characteristics affirm that the fabrication procedures did not affect the adsorptive and sensitive qualities of the raw carbon material that promote its use toward dopamine electrochemical sensing. The maintained noise levels confirm that the submicron- to micron-level thick parylene insulation remains intact and free of perforation that could amplify background fluctuations and resulting noise. Biodissolvable PEG (6000 g mol^–1^) (Sigma-Aldrich, 81260) was heated until liquid (hot plate at 80 °C) and applied onto the probes placed above a releasable glass substrate. The liquid was cooled to room temperature to form a rigid 0.5–1 mm thick shuttle that could be used to secure mechanically the ultra-small and compliant probes as they were inserted into the brain.

### Electrochemical FSCV methods

Dopamine signals were recorded using FSCV by applying a triangular waveform ramping from −0.4 to 1.3 or 1.4 V at a scan rate of 400 V/s and a cycle frequency of 10 Hz. The applied potential was held at −0.4 V between scans. A potential offset was applied in vivo in the range of 0.1 to 0.3 V, mainly to compensate for the shift in electrode potential of the implanted Ag/AgCl reference. The FSCV parameters were fixed for each subject. Prior to recording, the µIPs were conditioned for 15 min by applying the same triangular waveform at a cycle frequency of 60 Hz. The signals were collected using an in-house system^15^ with a noise floor of <0.1 nA, dynamic range of ±2000 nA, and sampling rate of 214 samples per scan. Implanted sensors were defined as nonfunctional if they had current noise greater than 1 nA or current saturation (i.e., magnitude of background current at any potential ≥2000 nA or limits of transducer range), both of which indicate a perforated or damaged insulation (Supplementary Fig. 2), or if a background current was <100 nA, which suggests a mechanical break of the carbon fiber^15,29^.

### In vitro flow-cell measurements

The µIPs and CFE sensors were tested in vitro for comparison of chemical recording performance based on their sensitivity and noise level parameters in order to characterize the effect of PEG application on the probes, and to create standards for dopamine and pH used for PCA of in vivo measurements. These measurements were done using a flow-cell apparatus with aCSF solvent prepared to a pH of 7.4 ± 0.05 to match a typical brain environment. pH standards were measured by flowing solutions (aCSF prepared at the physiological pH range of 7.2–7.6) at 0.05 intervals past the testing electrode in a bath of aCSF (pH of 7.4) and by recording subsequent changes in current. pH was adjusted by adding small amounts (<100 µL) of sodium hydroxide or hydrochloric acid to a 2 L aCSF solution and subsequently measured for each titration (VWR, sympHony benchtop meter). Dopamine hydrochloride (Sigma-Aldrich, H8502) was added to the aCSF to measure probe dopamine sensitivity at physiological levels of 0.25, 0.5, and 1 µM. Each device was evaluated three or four times at each condition and analyte concentrations with two separately prepared solutions of analyte. A syringe or gravity pump was used to drive solutions across the sensor. A sample injection valve (Valco Instruments, Model 22Z) or stopcock valve was used to switch flowing solutions. Devices were conditioned at 60 Hz for ~5 min prior to testing. The µIPs were shown to have similar sensitivity and correlations to levels of dopamine oxidation current (51.96 nA µM^–1^, *R* = 0.9999, *n* = 96 measurements from 4 probes) as those of the CFEs (37.74 nA µM^–1^, *R* = 0.9973, *n* = 60 measurements from 3 probes) over a range of physiological dopamine concentrations (0.25–1 µM) (Fig. 1c). Normalized sensitivity was calculated by taking the slope of the measured oxidation current divided by the background current as a function of dopamine concentration (Supplementary Fig. 4b, c)^15^. The noise of the probes was calculated by taking the root mean square of the background current or PCA-computed dopamine fluctuations during their voltammetric operation to assess their LOD and overall stability. Measurements of PEG effects on sensitivity and noise were made from four µIPs before and three µIPs after PEG coating. The PEG was dissolved in aCSF at room temperature prior to flow-cell measurements. All measurements of probe performance are reported in the Results as mean ± standard deviation and *P*-values are obtained by unpaired two-tailed *t*-test.

### Chemometric analysis

Background-subtracted data (Fig. 2d and Supplementary Figs. 2, 3 and 8) were generated by concatenating measured current at the probe for each voltage scan (*y*-axis) as successive time points (*x*-axis) to display voltage dependent current changes (nonlinear color scale). Dopamine produces current at selective reduction and oxidation (redox) potentials of –0.2 and 0.6 V for the implemented FSCV parameters^17,18^. Current changes also occur outside these redox potentials due to electrochemical reaction of other molecules, including those producing changes in pH^7,11,37,40^. PCA was implemented to dissociate current contributions of targeted dopamine molecules from pH and background current drift, and to compute the apparent change in concentrations of dopamine based on in vitro flow-cell calibration data^15,18^. All analyses were done in MATLAB (Mathworks, Matlab 2017a) as previously reported^6^. Comparisons of recorded dopamine concentrations between animals and across probes within a subject were made using one-way ANOVA.

### Surgical implantation of probes

All procedures involving animals were approved by the Committee on Animal Care at the Massachusetts Institute of Technology and were conducted in accordance with the U.S. National Research Council Guide for the Care and Use of Laboratory Animals. Nine Sprague-Dawley male rats (Taconic, 350–450 g, estimated age of 11–14 weeks at arrival) were used for probe implantation. Seven were implanted with µIPs, and two were implanted with CFEs. They were pair-caged pre-implantation and were individually caged post-implantation, with ad libitum food and water. Rats were anesthetized (1.5–2.0% isofluorane, 1 L min^–1^ oxygen) and administered an analgesic (Meloxicam, 2 mg kg^–1^) subcutaneously. The skin overlying the calvarium was incised and retracted. Craniotomies were stereotaxically guided (Stoelting, 51600, 51449) to place sensors in the right striatum (anteroposterior [AP] +1.5 mm, mediolateral [ML] +2.1 mm), a reference electrode in the contralateral hemisphere (AP –2.3 mm, ML –3.3 mm), and 2–3 stimulation electrodes in the MFB ipsilateral to the recording probes (AP –4.1 mm, ML +1.7 mm). Bone screws (Stoelting, 51457) were installed along the perimeter of the opening in the calvarium to secure the implanted devices for chronic use. A bare stainless steel wire (A-M Systems, 792900) was wound around 2 or 3 screws to serve as an electronic ground connection in some rats. Implant devices were then secured to the skull by cementing (Ortho-Jet, 0206) them to their neighboring screws. The dura mater was removed from the tissue above the striatum and MFB with the bent tip of a 32G hypodermic stainless steel needle.

The µIPs were implanted by lowering non-PEG coated probe tips with a micromanipulator at a rate of ~100–500 µm min^–1^. Basal parts of the PEG that secured the probe segments to be inserted were dissolved in 200–500 µm increments as the shuttle approached the brain surface by application with warm (35–38 °C) 0.9% saline. The probes were lowered incrementally until they reached a depth of dorsoventral (DV) 4.5–5.5 mm. CFE sensors (an array of 4 spaced ~500 µm apart) were implanted without PEG toward the same coordinates and with a similar insertion speed.

Reference Ag/AgCl electrodes were made of insulated silver wires (A-M Systems, 787000 and 786000) that were exposed 0.5–1 mm at the tip and were chlorinated in bleach overnight. These were placed above the exposed cortical surface (330 µm diameter wires) or penetrated 0.5 mm (178 µm diameter wires) into the contralateral hemisphere. The stimulation electrodes were 2 or 3 Pt/Ir electrodes (75–125 µm diameter) spaced 300–600 µm apart. The tip impedances ranged 2–1000 kΩ. These electrodes were lowered to an initial depth of DV 6.0 mm and then incrementally (100–200 µm) to the optimal depth (DV 6.0–8.7 mm) where stimulation was observed to produce the highest amplitude of dopamine release as measured by implanted striatal sensors. All implanted devices were connected to metal sockets (Mill-Max, 853-43-100) to provide connection for FSCV on subsequent days of recording. All devices were covered in cement so that only the sockets were exposed.

### MFB electrical stimulation

Rats were anesthetized (1.5–2.0% isofluorane, 1 L min^–1^ oxygen) and connected to FSCV instrumentation in each recording session. Current was delivered to the implanted MFB electrodes in a train of 24, 48, or 72 biphasic pulses with a fixed frequency of 60 Hz and pulse width of 2 ms) through a stimulus isolator (World Precision Instruments, A365) as triggered by the recording software. These parameters were derived from commonly reported MFB stimulation paradigms in rodents^4–6,8,9,12,39^. A variable number of pulses and amplitude (200, 250, or 300 µA) were used, and these parameters were usually fixed across sessions and for each subject (Supplementary Data 1).

### Histology

Electrolytic lesions were made at probe tips by applying cathodal current of 15 µA for 5 s. Brains were dissected from deeply anesthetized rats that were transcardially perfused with 0.9% saline followed by 4% paraformaldehyde (PFA) in 0.1 M phosphate buffer. Brains were then post-fixed by storing them in glass vials in 4% PFA overnight with gentle rocking at 4 °C. PFA solution was replaced by a 25% glycerol cryoprotectant solution in 0.1 M phosphate buffer with 0.1% sodium azide (Sigma, 438456) until the brain sank (overnight) at 4 °C. The brains were stored in the same solution at 4 °C until sectioning. The brains were then frozen in dry ice, and were cut at 30 μm thickness in the horizontal plane on a sliding microtome. Sequential sections were placed individually into adjacent wells of a multi-well plastic tray containing 0.1% sodium azide in 0.1 M phosphate buffer for storage at 4 °C until the immunostaining procedure.

### Histochemistry

Sections were rinsed three times for 2 min in 0.01 M phosphate buffer saline (PBS) containing 0.2% Triton X-100 (Tx) (Sigma-Aldrich, T8787) and then were incubated in PBS-Tx with 0.5% of tyramide signal amplification (TSA) blocking reagent (PerkinElmer, FP1012) for 20 min. The sections were incubated with primary antibody solutions containing chicken anti-GFAP (Abcam, ab4674) [1:1000], rabbit anti-Iba1 (Wako, 019-19741) [1:2000] or rabbit anti-tyrosine hydroxylase (TH) (Abcam, ab112) [1:4000], and/or goat anti-mu-opioid receptor 1 (MOR1; Santa Cruz, SC-7488) [1:500] in PBS-Tx with TSA blocking reagent for 24 h at 4 °C for primary incubation. The sections were then rinsed three times for 2 min in PBS-Tx, and then were incubated for 2 h in the secondary antibody solution containing goat anti-chicken Alexa Fluor (AF) 633 (Life Technologies, A-21103) [1:300], goat anti-rat AF 546 (Life Technologies, A-11081) [1:300], goat anti-rabbit AF 488 (Life Technologies, A-11034) [1:300], and/or donkey anti-goat AF 647 (Life Technologies, A-21447) [1:300] in TSA blocking reagent in PBS-Tx. The secondary antibody solution contained donkey anti-chicken FITC (Abcam, ab63507) [1:300], donkey anti-rat 550 (Life Technologies, SA5-10027) [1:100], donkey anti-goat 647 (Life Technologies, A-21447) [1:300], and/or donkey anti-rabbit AF750 (Abcam, ab175731) [1:300] for identifying recorded sites inside of striatal defining boundaries (i.e., MOR1 or TH) and/or MFB expressing markers (i.e., TH). The sections were rinsed three times for 2 min in PBS-Tx, incubated for 2 min in DAPI (Life Technologies, D1306) [1:1000] in PBS, and then were rinsed three times for 2 min in 0.1 M phosphate buffer, mounted onto glass slides and coverslipped with ProLong Gold antifade reagent (Life Technologies, P36930).

### Histological evaluation of tissue damage and inflammation

Histochemical images were obtained on a scanning (TissueGnostics, TissueFAXS Whole Slide Scanning System) confocal microscope (Zeiss, LSM 510) with ×10 air objective. The location of probe tracks was determined in each horizontal slice by identifying holes that matched with expected probe configurations and geometries. Implant identification was straightforward for CFEs that produce a demarcated 70–90 µm diameter hole for most stains (GFAP, Iba1, IgG, and DAPI). µIPs required more procedures to locate because their appearance was seen to be similar to that of physiological blood vessels. µIP implanted sites were located by first identifying tip locations marked by electrolytic lesions that reproducibly produced pronounced changes in IgG and sometimes Iba1 and GFAP (Fig. 2b). These lesion coordinates were then used to identify the corresponding implant track at more dorsal (0.5–1 mm) planes to decouple the artificially generated inflammation induced by the electrolytic lesions made at probe tips. Channel (GFAP, Iba1, IgG, and DAPI) images of the same section were imported into a computer program (Matlab 2017a, Mathworks) to quantify fluorescent intensity changes of the individual channels as a function of distance from the implanted probe. The center of the probe shaft was identified manually on the image, and this same origin was used for all other channels of the same slice for analysis. Pixel intensities were averaged in 2 µm bins from the probe origin to 500 µm away across radial lines at 0.5° intervals. Averaging was performed over a 90° arc around the probe manually selected to avoid anatomical boundaries and neighboring probes. Projection intensities were divided by the background intensity calculated by averaging intensities over a region 600–620 µm away from the origin. These normalized fluorescence intensity values were computed over at least three horizontal sections per implanted probe (*n* = 4 CFE and *n* = 4 µIP type devices). Two-tailed *t*-test was used to assess differences in normalized intensity values between groups.

## Electronic supplementary material

Supplementary Information

Supplementary Data 1

Description of Additional Supplementary Files

## Data Availability

The raw data generated and analyzed during the current study are available from the corresponding author upon reasonable request.
