# Supplementary material for: Cellular-scale probes enable stable chronic subsecond monitoring of dopamine neurochemicals in a rodent model
Source: Commun Biol. 2018 Sep 12;1:144. doi: 10.1038/s42003-018-0147-y (PMC6135761; doi:10.1038/s42003-018-0147-y)
Supplement: Supplementary file 1 — Supplementary Information [file 42003_2018_147_MOESM1_ESM.pdf]

## Supplementary Figures

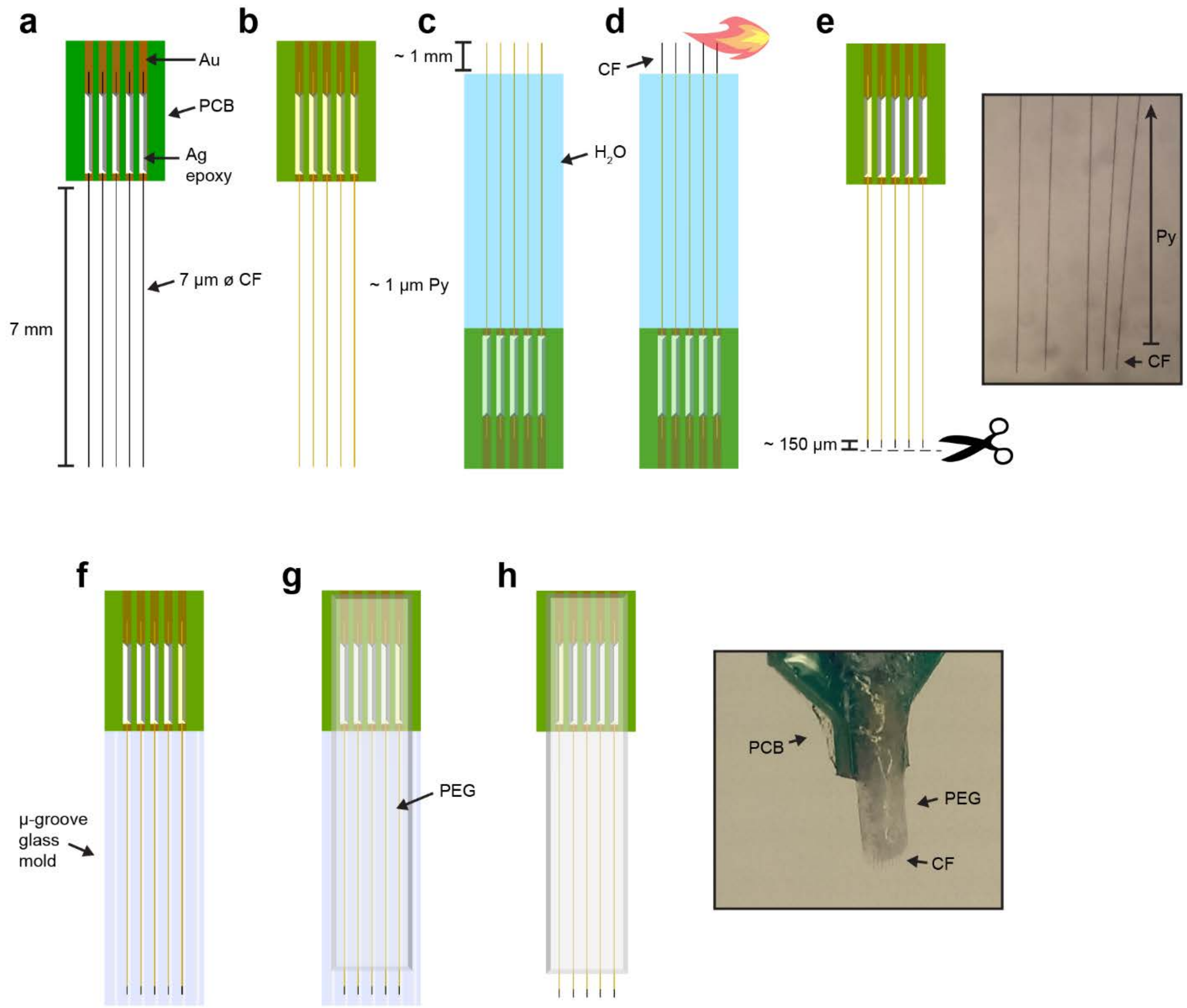


**Supplementary Fig. 1** Fabrication of implantable probes. **a** Array of bare 7-μm diameter carbon fibers (CFs) bonded to gold electrical interconnects (250 μm pitch) on a printed circuit board (PCB) with silver epoxy. **b** Parylene (Py) conformally deposited all over device with a thickness of ~1 μm. **c** Array submerged into water for thermal insulation while tips to be patterned are exposed ~1 mm above the water. **d** Butane torch used to strip parylene and reveal underling CF. **e** Carbon fiber tips trimmed to a length of ~150 μm with fine scissors (left) and photo of trimmed probes (right). **f** Probes aligned atop a μ-groove glass mold. **g** Molten PEG applied to array and solidified at room temperature. **h** Array detached from mold (left) and photo of completed PEG coated array (right).

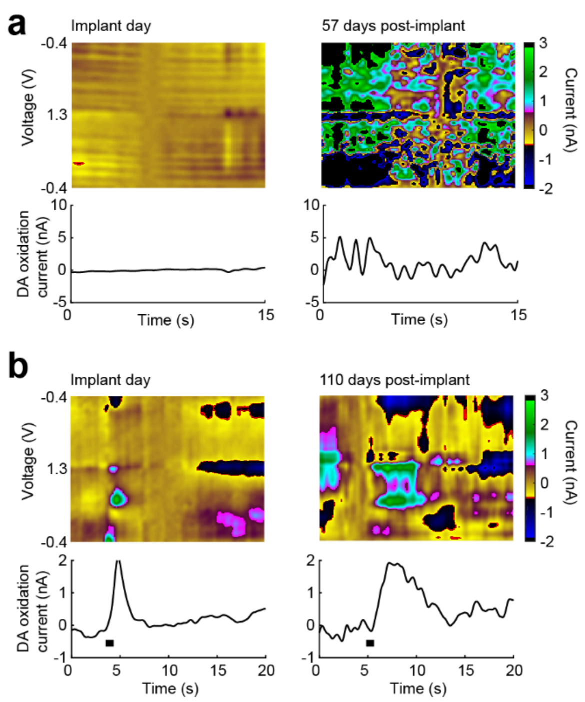


**Supplementary Fig. 2** Cases of degraded performance of implanted lift-off fabricated probes over time. **a** Background-subtracted current across applied voltage plotted in color scale at right (top), and dopamine (DA) oxidation current (bottom) recorded from cl042. Noise increased from 0.011 nA on the implant day (left) to 0.397 nA on post-implant day 57 (right). **b** Same as in **a** for another probe (cl093) implanted in a different rat. Noise increased from 0.018 nA on the implant day to 0.354 nA on post-implant day 110. Time of MFB stimulation indicated by black bars.

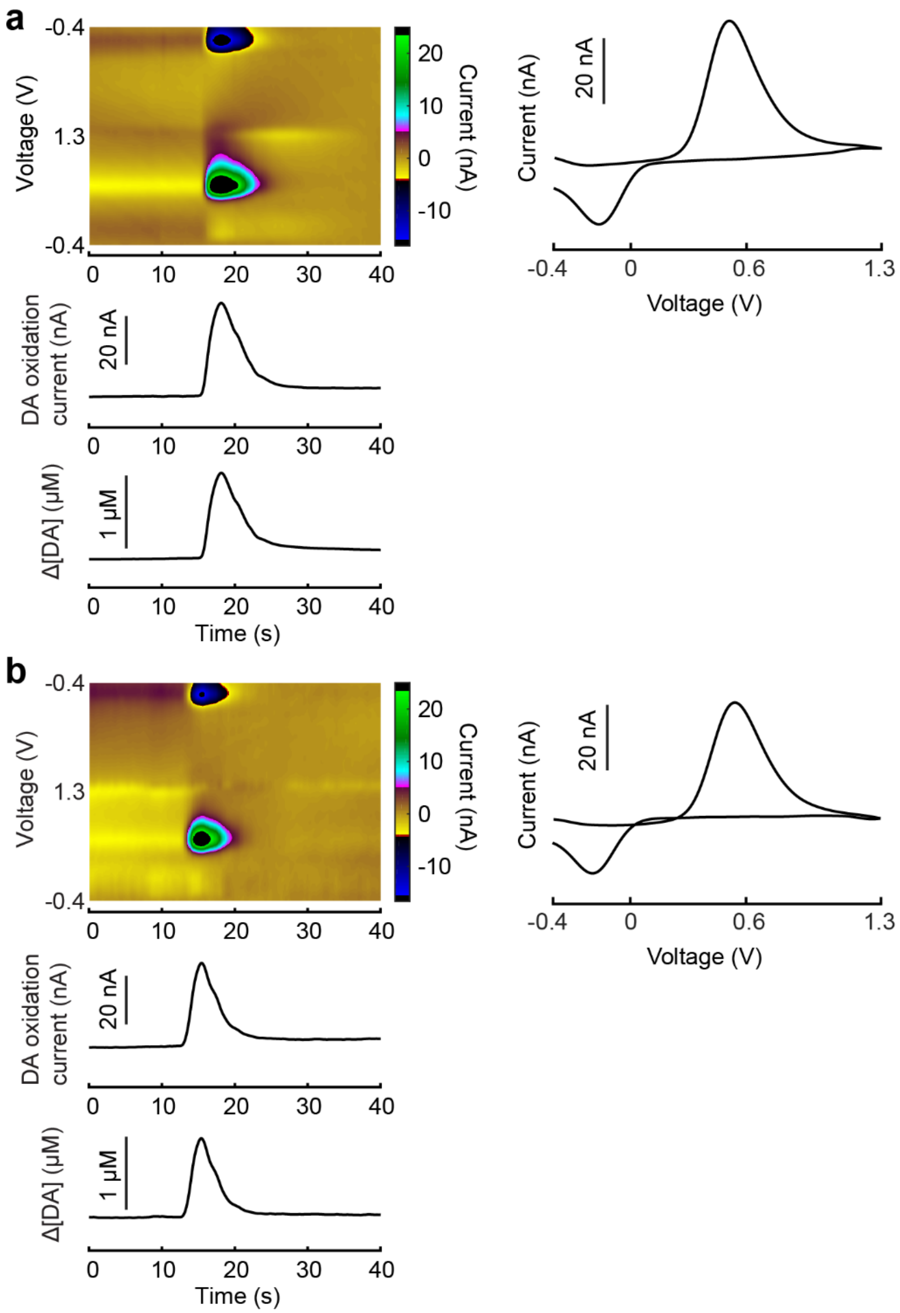


**Supplementary Fig. 3** *In vitro* flow-cell measurements from a µIP and a standard CFE for a 1 µM dopamine (DA) bolus injection in aCSF. Background-subtracted current (top left), current at dopamine oxidation potential (i.e., dopamine oxidation current; middle left), PCA-extracted dopamine concentration change (Δ[DA]) (bottom left), and cyclic voltammogram (current vs. voltage; top right), recorded by a µIP (**a**) and by a CFE (**b**).

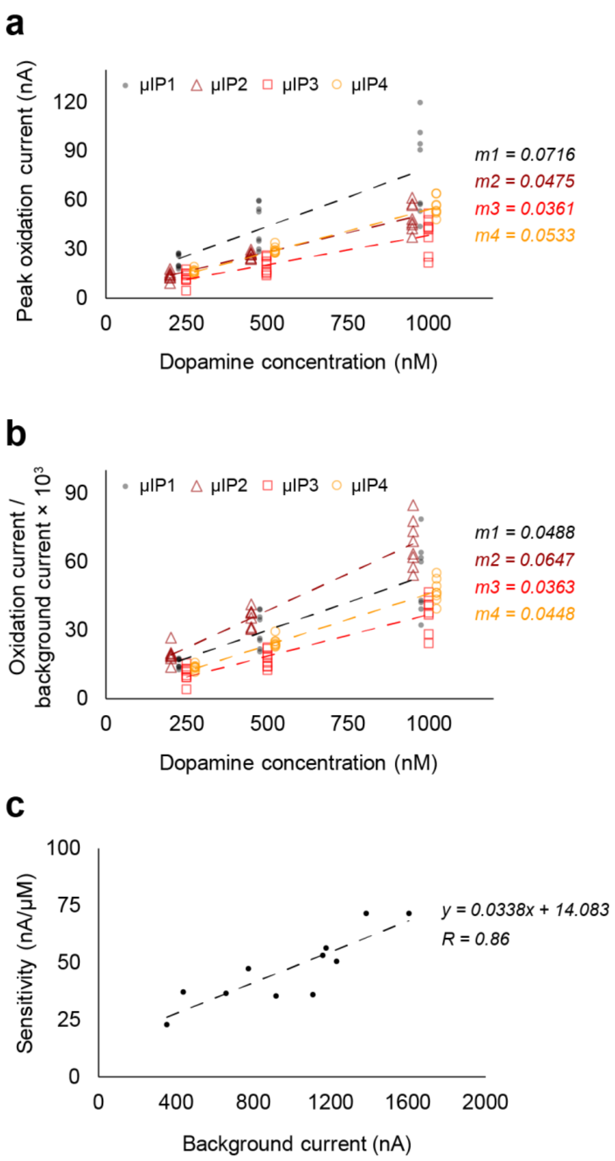


**Supplementary Fig. 4** *In vitro* measurements of peak oxidation current as a function of dopamine concentration. **a** Peak oxidation current vs. concentration for 4 µIPs (left) and 3 standard CFEs (right) measured in flow cell. All measurements were taken at prepared concentrations of 250, 500 and 1000 nM, but data are splayed along x-axis to visually discriminate data for different devices. Slope (*m*) (i.e., non-normalized sensitivity, nA $nM^{-1}$) of fitted curves displayed on the right of each plot. **b** Measured oxidation current normalized to probe's apparent capacitance (i.e., divided by background current and multiplied by 1000), displayed as in **a**. **c** Sensitivity of measured probes as a function of background current with linearly fitted curve. Its regression equation and Pearson's correlation coefficient (*R*) are shown on the right.

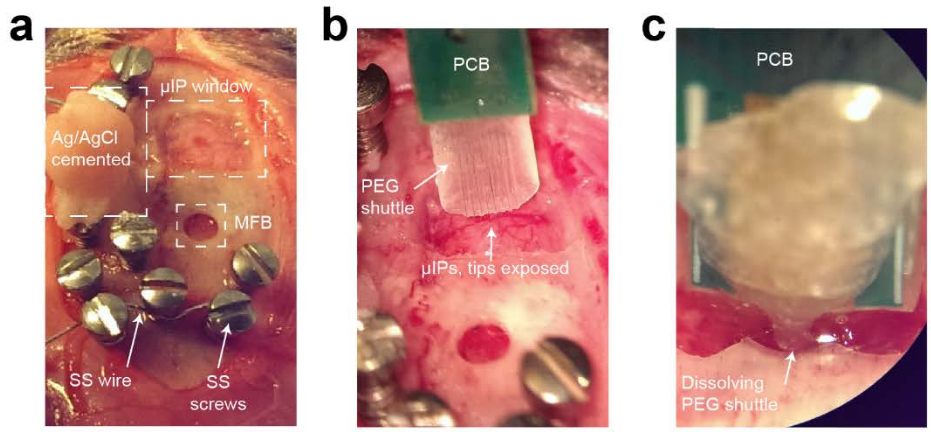


**Supplementary Fig. 5** Surgery and device implantation. **a** Implantation of Ag/AgCl reference electrode, and craniotomies for stimulating electrode and µIPs. Nine stainless steel (SS) screws were installed intracranially to secure devices by cement and provide electronic ground via wrapped stainless steel wire. **b** Printed circuit board (PCB) holding multiple µIPs placed above dura mater to be driven toward target striatal regions. PEG shuttle encasing the µIPs remains intact, and µIP tips can be seen recessed below PEG base. **c** µIPs driven ~4 mm deep into brain tissue with overlying PEG almost completely dissolved.

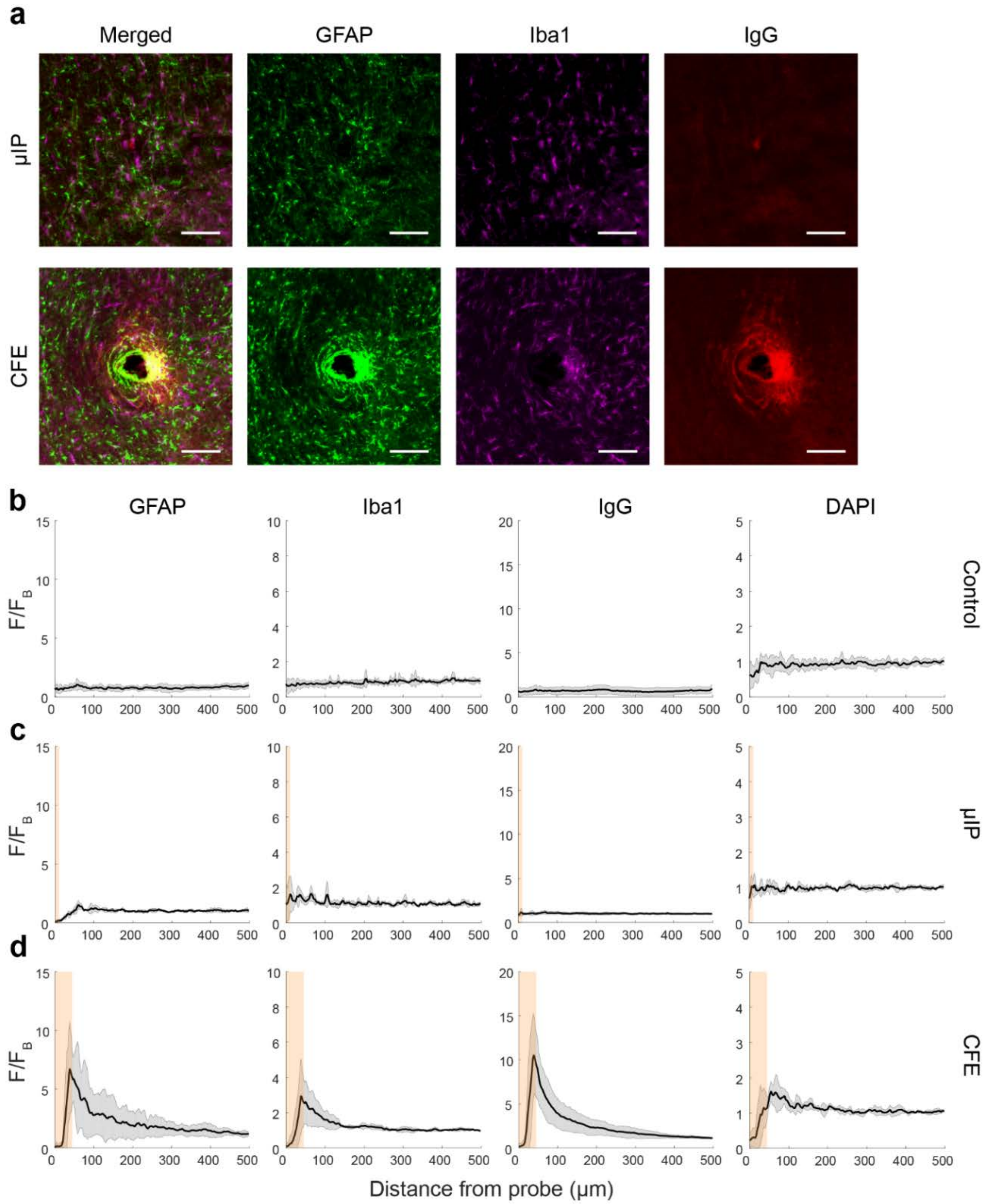


**Supplementary Fig. 6** Tissue response induced by chronically implanted µIP and CFE. **a** Confocal microscope images of horizontal sections through striatum immunohistochemically stained for markers of inflammation, including astrocytes (GFAP), microglia (Iba1), and blood brain barrier permeability (IgG), around sites of implanted µIP and CFE shafts at DV ~3.6 mm. Scale bar: 100 µm. **b–d** Fluorescence intensity profile of cellular nuclei (DAPI), GFAP, Iba1, and IgG as a function of distance from implanted probe for unpenetrated (**b**), µIP (**c**), and CFE (**d**) (each averaged from 4 chronically implanted probes in 2 rats). Gray shading shows ± SD. Tan shading denotes estimated location of implanted probe.

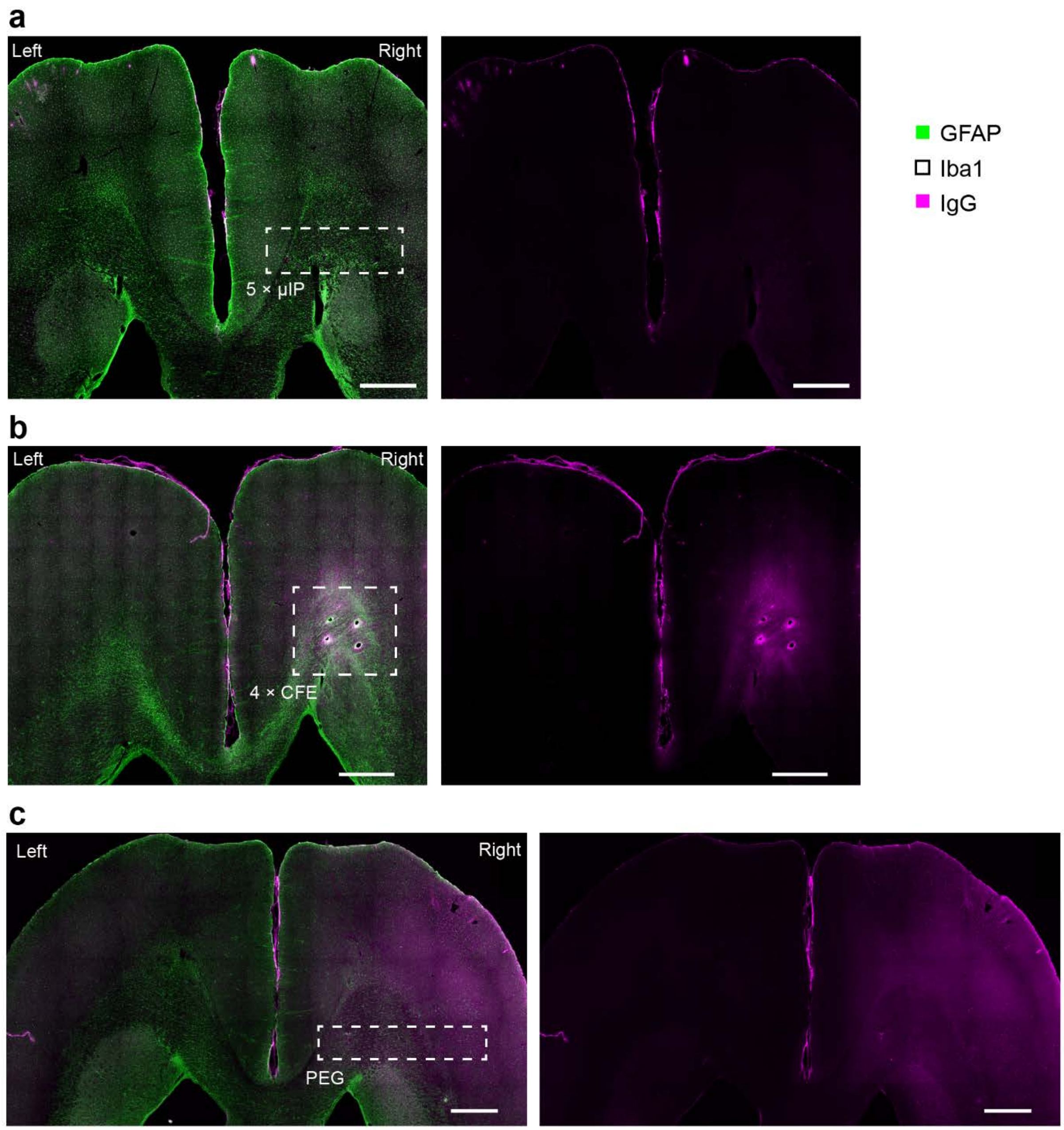


**Supplementary Fig. 7** Images of brains implanted with µIPs, CFEs, or µIPs and undissolved PEG shuttle. **a** Five µIPs implanted without insertion of PEG shuttle (horizontal section of a brain harvested on post-implant day 95). Visible differences in inflammatory markers for astrocytes (GFAP), microglia (Iba1), and blood brain barrier permeability (IgG) (left) are not clearly distinguishable between hemispheres through regions implanted with µIPs. Right panel shows the IgG expression alone for the same image. **b** Four implanted CFEs (post-implant day 74) showing broad increases in all markers of inflammation. **c** Implanted PEG shuttle before its dissolution (post-implant day 141) showing broad increase in IgG. All horizontal sections were taken approximately at DV 3.2 mm. Scale bars represent 1 mm, dashed lines roughly indicate estimated boundary of device penetration, and color codes for inflammatory markers are displayed at the top right corner.

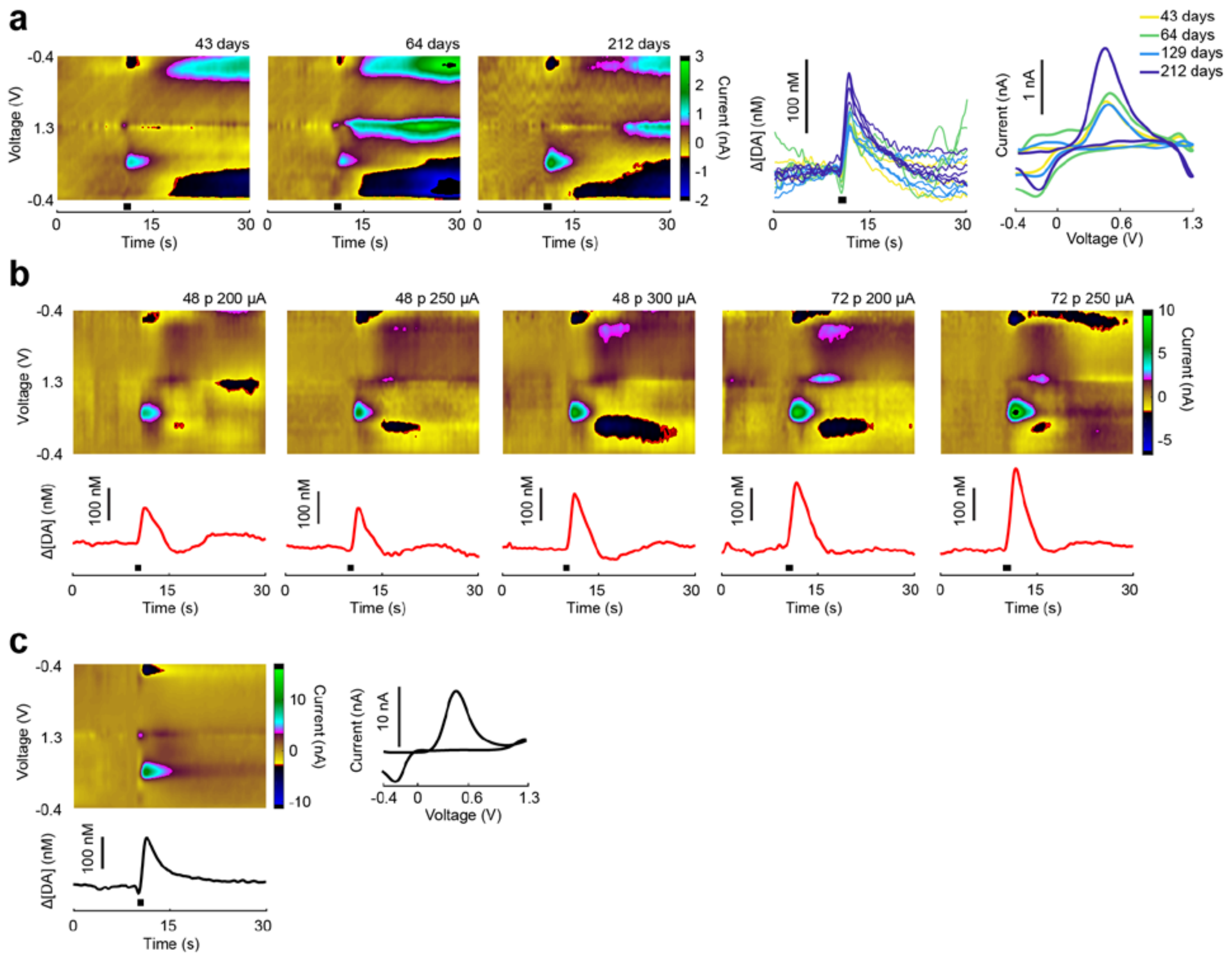


**Supplementary Fig. 8** Examples of neurochemical measurements from µIPs. **a** Chronic measurements of dopamine recorded from c123 with a fixed stimulation parameter on post-implant days 43, 64 and 212. **b** Dopamine recorded from c201 on post-implant day 74, as a function of stimulation intensity. **c** Acute measurement of dopamine from c202. MFB stimulation occurred at 10 s (indicated by black bars).

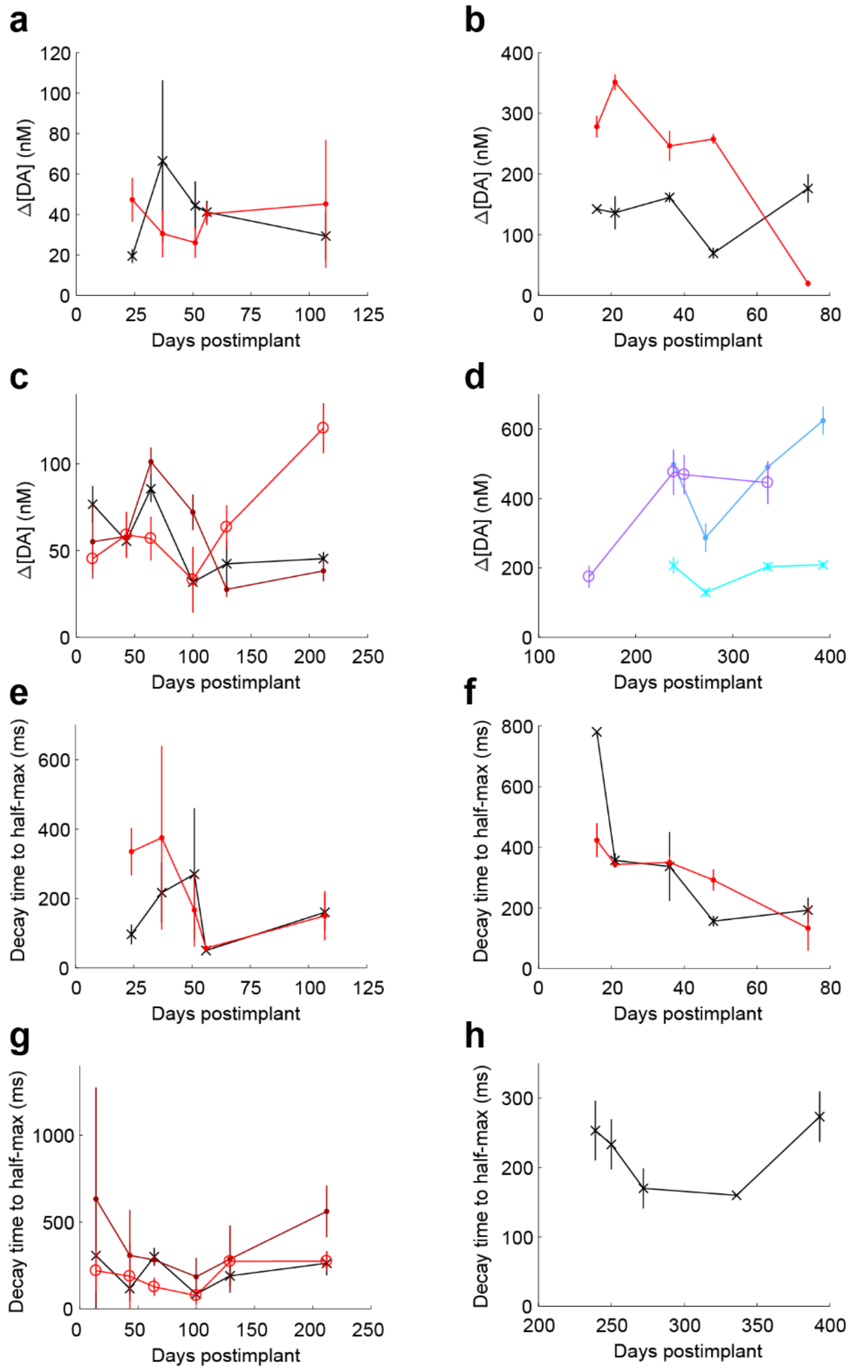


**Supplementary Fig. 9** Longitudinal measurements of dopamine from chronically implanted µIPs. **a–c** Evoked dopamine (DA) release over time measured from a set of probes c171 (**×**) and c172 (•) (**a**), c201 (**×**) and c202 (•) (**b**), and c121 (**×**), c122 (•), and c123 (○) (**c**), each in a different rat and with a fixed stimulation parameter. **d** Measured dopamine over time for a single probe (c081) in a rat for 3 different stimulation intensities: low (300 µA and 24 pulses) (**×**), medium (200 µA and 48 pulses) (•), and high (250 µA and 48 pulses) (○). **e–h** Measured decay time from maximum to half-maximum evoked DA for same probes and parameters as in **a–d**. Data for the medium stimulation are shown in **h**. Error bars represent 95% confidence intervals (i.e., ± SEM × 1.96).

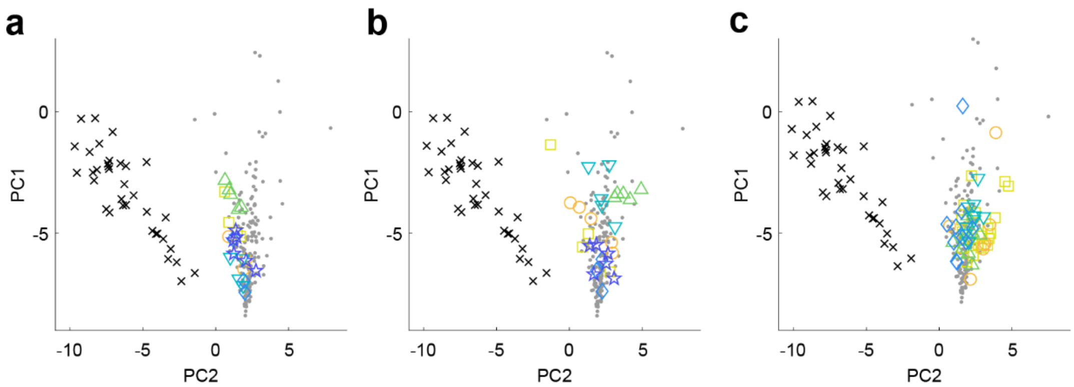


**Supplementary Fig. 10** Principal component analysis of chronic measurements from individual probes across time. Principal component scores (PC1 and PC2) over time for measurements made at 14 (□), 43 (○), 64 (Δ), 100 (▽), 129 (◊), and 212 days (☆) post-implant from two implanted probes, c121 (**a**) and c122 (**b**), implanted in the same rat, and at 24 (□), 37 (○), 51 (Δ), 56 (▽), and 107 days (◊) post-implant from another probe, c172, in another rat (**c**), overlaid with *in vitro* dopamine (•) and pH (×) standards, as shown in **Fig. 5**.
