# Supplementary material for: Cellular-scale probes enable stable chronic subsecond monitoring of dopamine neurochemicals in a rodent model
Source: Commun Biol. 2018 Sep 12;1:144. doi: 10.1038/s42003-018-0147-y (PMC6135761; doi:10.1038/s42003-018-0147-y)
Supplement: Supplementary file 3 — Description of Additional Supplementary Files [file 42003_2018_147_MOESM3_ESM.pdf]

Description of Additional Supplementary Files

File Name: Supplementary Data 1
Description: Parameters used for testing implanted μIPs
